# Perceived Epidemic Risk and Depression Symptoms during the COVID-19 Pandemic: The Mediating Role of Security and the Moderation Role of Perceived Discrimination

**DOI:** 10.3390/ijerph19127054

**Published:** 2022-06-09

**Authors:** Yunjun Hu, Lingling Shu, Huilin Zhang, Chen Wang, Chengfu Yu, Guanyu Cui

**Affiliations:** 1 Department of Students’ Affairs, Wenzhou University of Technology, Wenzhou 325000, China; 20180114@wzut.edu.cn; 2 School of Education, Wenzhou University, Wenzhou 325035, China; 18210452123@stu.wzu.edu.cn (L.S.); 21450408025@stu.wzu.edu.cn (H.Z.); 3 Center for Brain, Mind and Education, Shaoxing University, Shaoxing 312000, China; 2020000101@usx.edu.cn; 4 School of Education, Guangzhou University, Guangzhou 510006, China; yuchengfu@gzhu.edu.cn

**Keywords:** perceived epidemic risk, depression symptoms, security, perceived discrimination, mediated moderation model

## Abstract

The 2019 coronavirus disease pandemic has resulted in a significant increase in the incidence and prevalence of mental health problems such as anxiety and depression, posing a threat to peoples’ lives and health safety all over the world. Research suggests some potential relationships among perceived risk, discrimination, security, and depression symptoms. However, little attention has been paid to the complex mechanisms of the associations between these variables. This study aimed to examine the mediating role of security and moderation role of perceived discrimination in the prediction of perceived epidemic risk on depression symptoms. Thus, we aimed to identify if perceived epidemic risk is a positive predictor of depression. A cross-sectional study was conducted through an anonymous online survey in China during the COVID-19 pandemic which measured perceived epidemic risk, discrimination, security, and depression symptoms. A total of 3443 valid questionnaires were obtained. The results indicated that depression symptoms were predicted by perceived epidemic risk through the mediating role of security, and this mediating role of security was moderated by perceived discrimination. Specifically, high levels of perceived discrimination may lead to a significant decrease in personal security, thus clustering depressive symptoms. These findings shed light on the influence of the perceived risk of the epidemic on depression symptoms in the context of the epidemic situation, which may help to develop targeted interventions.

## 1. Introduction

The world is facing an unprecedented challenge with the growing COVID-19 pandemic; the disease is highly infectious and has become one of the most important threats to human life and health. Sudden public crisis events can easily cause psychological reactions such as tension, anxiety, and even panic, which may lead to psychological disorders such as stress disorder and depression [1]. Risk perception is an individual’s subjective judgment of risk in a crisis event, including uncertainty of the severity of the threat and subsequent consequences [2]. Individuals’ perceived epidemic risk often causes great psychological stress [3], reduces their sense of security, increases discrimination against the individuals and groups concerned, and induces or even aggravates depression. With the increase in the COVID-19 infections and deaths, COVID-19-associated stigma has been identified as an important barrier to controlling its transmission [4]. Stigma has been associated with a higher risk of depressive symptoms during the COVID-19 pandemic [5]. Perceived discrimination is an essential public health problem. Feeling discriminated against is associated with poor psychological and physical health outcomes, including increased depression, anxiety, hypertension, and mortality [6,7]. In addition, a negative relationship between perceived discrimination and health has been shown in cross-sectional and prospective studies [8]. In order to protect people’s mental health, a series of preventive measures should be taken to reduce people’s perceived increased risk [9]. Although previous studies have focused on some of these issues, so far, there has been little research on the interactive mechanisms of the above variables, which has affected our understanding of the relationship between these variables and may even contribute to depression levels in relevant individuals and groups during the epidemic. Therefore, the current study aims to explore the role of individuals’ perceived discrimination and security in line with their perceived epidemic risk and depression symptoms.

### 1.1. The Association of Perceived Epidemic Risk and Depression Symptoms

The concept of “perceived risk”, derived from RISP (Risk Information Seeking and Processing) refers to the subjective judgment of risk characteristics, severity, and management [10]. The latest research results have found that epidemic risk may affect individual mental health in the early stage of public health emergencies [11]. The epidemic risk of COVID-19 is associated with individuals’ depression symptoms. From a study result in Pakistan, 33%, 40%, and 27% of the participants were found to experience depression, anxiety, and stress, respectively. Among them, 52% of the participants showed mild to very severe levels of depression, anxiety, and stress disorders [12]. In one study in Spain, 12.7% of 692 participants showed possible rates of depression caused by worry about disease, behavior restriction, and changes in the economic environment [13]. In one study in America, among 4852 participants during the period of the COVID-19 pandemic, 64% felt hopeless, down, or depressed [14]. Scholars have found that the epidemic has caused citizens to be overwhelmed by psychological problems such as fear, anxiety, and depression [15]. In addition, a Greek study found that 9.31% of people had clinical depression and 8.5% had severe depression [16]. Furthermore, lesbian, gay, transgender, queer, intersex and other sexual and gender minorities are more vulnerable to discrimination and adverse mental health outcomes [17].

According to Lazarus’ transactional model of stress, the stress response produced by stimulation to an individual often depends on the cognitive processing of stimulation signals in the individual brain. When an individual is threatened by stimulation, he or she will assess whether the stimulation is harmful and whether their coping skills are sufficient. When individuals appraise that they can deal with the stressor they will perceive it as challenging; on the contrary, when they cannot deal with the stressor, they will perceive it as distressing [18]. The impact of the epidemic situation on individual mental health may depend on individual perception of epidemic risk. The higher the level of risk perception, the greater the psychological pressure placed on people [19]. Increased stress is associated with many mental health disorders such as anxiety and depression [20,21,22]. Many research results have shown that epidemic risk cognition has a significant positive impact on anxiety and depression [3,23,24,25]. Therefore, we hypothesized that individuals’ perceived epidemic risk would have a positive predictive effect on their depression.

### 1.2. The Mediating Role of Security

When an individual encounters a serious disaster, the need for security is particularly obvious. Security is the sense of feeling, which is separated from fear and anxiety, containing confidence, safety, and freedom; an individual feels safe especially when his needs are met at present or in the future [26]. Psychological security is a subjective feeling at an individual level, a subjective perception of the dangers or risks that may appear around and threaten the body and mind, and a perception of whether one has the ability to deal with the dangers or risks [27]. Under the stress of the epidemic situation, peoples’ sense of security has significantly decreased, and an individual may consider security issues very seriously [28]. According to Davies and Cummings’ (1994) theory of emotional security, an individual emotional and psychological needs comprise being taken care of, being protected, and being free from troubles [29]. In a threat situation, when the stimulation an individual receives exceeds his limit of self-control and release of energy, he will immediately realize the threat or possible negative consequence of the stress to his well-being through cognitive representation, and experience fear, hostility, and other negative emotions.

Security feelings are highly associated with psychological pain. Researchers have found that a low level of security is an important indicator of mental health problems [30]. Moreover, a lack of security will reduce self-efficacy, and thus affect an individual’s psychological and behavioral adaptation in the environment [31]. The latest research has showed that stressors related to the novel coronavirus will frustrate employees’ sense of belonging, increase their sense of insecurity, and have a negative impact on their behavior [32]. Because the epidemic is uncontrollable and unpredictable [33], this increases citizens’ sense of insecurity, fear of infection, and social isolation caused by lockdowns [34,35,36]. Depression, a common psychological problem with high prevalence, is a high-risk factor affecting personal health and well-being [37]. In the related research on attachment and depression, it is found that safe attachment is conducive to the high self-esteem and effective emotion regulation of adolescents. Adolescents with safe attachment have stronger adaptability, and continuous safe attachment has a good protective effect on adolescent depression [38]. Security has been found to play a mediating role between stimulus events and adolescent depression [37], so we assume that security may mediate the association between perceived epidemic risk and depression.

### 1.3. The Moderating Role of Perceived Discrimination

Because of the discrimination and prejudice against COVID-19, patients, as well as their family members and neighbors, are under enormous pressure. Perceived discrimination refers to an individual’s perception of unfair treatment due to belonging to a specific social category [39]. According to the theory of Spencer’s (1995) phenomenological variant of ecological systems, when facing the pressure of a new environment, stigmatized group members will form new coping strategies [40]. If these strategies are valuable for self-development, they will be preserved and gradually form the individual’s identity, which will either have a negative impact or constructive effect [41].

Discrimination brings potential harm to people’s development. A study conducted by Schaeffer (2019) found that discrimination perception has obvious negative effects on individual psychological development and social adaptation [42]. As a source of pressure, perceived discrimination will reduce individual well-being, reduce mental and physical health, weaken self-confidence, and render individuals at a disadvantage in competition. Moreover, it has been widely documented to increase pain, including depression and anxiety [8]. Other studies have found that groups experiencing high levels of discrimination for a long time experience poor physical and mental health [43]. For example, weight discrimination increases the risk of dementia by up to 40 percent [44]. Moreover, assessing the discriminatory experience of older Puerto Ricans could prevent depression and cognitive decline in this population [45].

Other studies have found that discrimination perception has adverse effects on individual mental health and social function. Perceived discrimination was found to increase depressive symptoms of mainland immigrants in Hong Kong [46]. A study by Weeks and Sullivan (2019) which analyzed survey data of children’s health from 60,700 children and adolescents aged 6–17 found that experiencing racial discrimination significantly increased the incidence of a series of mental health problems such as depression, anxiety, and behavioral disorders [47]. In the longitudinal study of Hackett (2019), it was also found that perceived discrimination can lead to increased psychological distress, decreased mental function, and decreased life satisfaction [48]. Furthermore, measures to reduce the discrimination suffered by international students may improve their mental health [49]. On the contrary, some researchers have suggested that perceived discrimination has a positive impact on mental health. For instance, Bourguignon et al. (2006) found that perceived discrimination had a positive effect on group self-esteem and protected individual self-esteem to a certain extent [50]. Moreover, Tyler et al. (2021) found discrimination increased the prosocial behavior of some groups [51]. Nevertheless, discrimination perception may play a moderating role between perceived epidemic risk and depression.

### 1.4. Current Study

**Figure 1:**
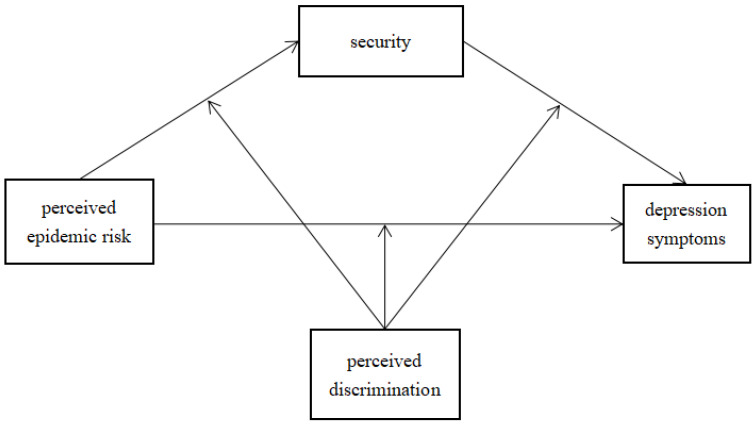
The proposed moderated mediation model.

To fill the gap in understanding the effect of perceived epidemic risk on depression, the current study constructed and tested a moderated mediation model (see Figure 1). Specifically, we examined the relationship between perceived epidemic risk and depression and extended previous research by investigating the mediating effect of security and the moderating effect of perceived discrimination on this relationship. We proposed the following three hypotheses:

**Hypothesis** **1.** *Perceived epidemic risk has a positive predictive effect on depression.*

**Hypothesis** **2.** *Security plays a mediating role between perceived epidemic risk and depression.*

**Hypothesis** **3.** *Perceived discrimination plays a moderating role among perceived epidemic risk, security, and depression.*

## 2. Materials and Methods

### 2.1. Participants and Procedure

Convenience sampling method was used and questionnaires were sent out nationwide and retrieved anonymously by SO JUMP, an online survey. After filtering and eliminating the invalid cases, 3443 (*N_males_* = 1225, *N_females_* = 2218) data samples were collected. Notably, 68.8% were under 25 years old, 6.8% were between 26 and 30 years old, 13.6% were between 31 and 40 years old, 8.1% were between 41 and 50 years old, and 2.7% were over 51 years old. Moreover, 68.6% of the participants had obtained at least a university degree. The proportions of non-medical students and medical students were 52.1% and 2.1%, respectively, and 45.8% of people were employed. Among them, 21.6% were from other professions, followed by 13.8% from science, education, culture and health. Moreover, 15.7% lived in provincial capital cities, 24.0% in prefecture-level cities, 19.0% in county-level cities, and 41.2% in towns and villages.

All participants (*M_age_* = 26.25 years; *SD* = 9.30) voluntarily completed the survey.

### 2.2. Measures

#### 2.2.1. Perceived Epidemic Risk

The perceived epidemic risk was measured by two items (i.e., “How likely is it that you or your family may be exposed to COVID-19 patients or potential patients” and “How likely is it that your life or your family’s life may be at risk from COVID-19”), which were similar to items used by Zhou (2021) [52] and Li and Lyu (2021) [3], and were adopted to measure participants’ perceived epidemic risk during the COVID-19 epidemic. We used a 5-point Likert scale for each item, ranging from 1 (totally impossible) to 5 (highly possible). Higher scores indicated higher perceived epidemic risk. Internal consistency in this sample was satisfactory (α = 0.79).

#### 2.2.2. Security

The “certainty control” subscale in the Chinese version of the security questionnaire was widely used to measure participants’ sense of control in the face of possible physical or psychological dangers or risks in China [27,53,54,55]. This questionnaire consisted of 8 items (i.e., “I feel life is always full of uncertainty and unpredictability”; reverse coded), and each item was used to measure certainty in control on a 5-point Likert scale, ranging from 1 (strongly disagree) to 5 (strongly agree). Higher scores indicated a higher level of security. This measure showed good internal consistency (α = 0.94) in this sample.

#### 2.2.3. Perceived Discrimination

Based on the “Experiences of Discrimination” (EOD) measure, three items (i.e., “How often do you feel excluded”, “How often do you feel threatened or intimidated” and “How often do you feel insulted or abused”) were selected and used to assess the perceived degree of discrimination/abuse in real life and online during the COVID-19 epidemic on a 5-point Likert ranging from 1 (never) to 5 (almost always) [56]. Higher scores on this measure indicated higher perceived discrimination. Cronbach’s alpha in this sample was satisfactory (α = 0.95).

#### 2.2.4. Depression Symptoms

Thirteen items of the depression subscale from the Symptom Checklist-90-Revised were used to assess participants’ degree of depression on a 5-point Likert scale for each item [57,58], such as “Feeling down in energy or slowed down” and “Thoughts of ending life”, ranging from 1 (not at all) to 5 (extremely). This scale is widely used in the world and has good validity [59,60]. Higher scores on this measure indicated a higher level of depression. This measure in this sample had good internal consistency (α = 0.94).

### 2.3. Data Analysis

SPSS 25.0 and Mplus 8.0 were used for data analysis. Before analyzing the data, we screened the questionnaire and eliminated 173 invalid data samples. Firstly, a preliminary descriptive statistical analysis and correlation analysis were conducted on the data with SPSS 25.0 to explore the correlations between perceived epidemic risk, security, perceived discrimination, and depression symptoms. Then, we used the ML Estimator to estimate the parameters of the Structural Equation Model (SEM) in Mplus 8.0 to perform the mediation analysis. Path analysis was used to examine the causal relationship between independent variables and dependent variables. Repeat sampling was performed with BC Bootstrap, and the test was conducted by estimating the 95% confidence intervals of the mediating and moderating effects through 1000 samples. If the confidence intervals did not contain zero, statistical significance was indicated [61]. Afterwards, the mediating role of security between perceived epidemic risk and depression symptoms was confirmed, and we also found a moderating role of perceived discrimination between security and depression symptoms. Ultimately, we reported the model fit indices assessed by the conventional levels of the goodness of fit [62,63]. The evaluation indicators selected in this study were the comparative fit index (CFI), the Tucker–Lewis index (TLI), the standardization root mean square residual (SRMR), and the root mean square error of approximation (RMSEA). When CFI and TLI were greater than 0.90 and SRMR and RMSEA were less than 0.08, the model was considered to fit well [63,64].

## 3. Results

### 3.1. Preliminary Analysis

**Figure 2:**
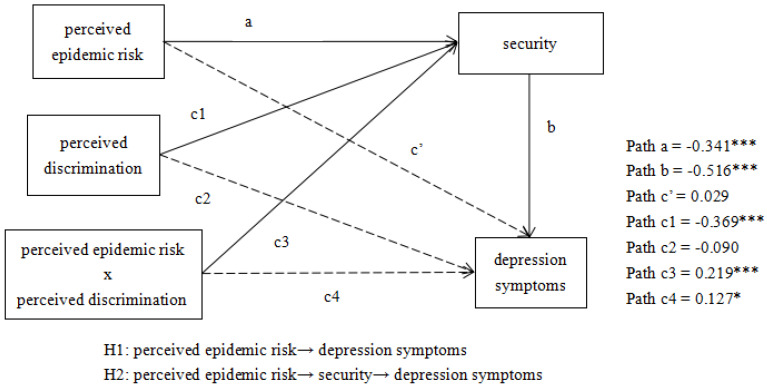
*N* = 3443; *p* > 0.05, * *p* < 0.05, *** *p* < 0.001; In this model, the indent effect of perceived epidemic risk on depression symptoms through security and the direct effect of perceived epidemic risk on security are supposed to be moderated by perceived discrimination.

After controlling for the influence of gender and age, which are not shown in Figure 2, we generated the fitting indices of the model: CFI = 0.993, TLI = 0.959, χ^2^/df = 7.046, RMSEA (90%CI) = 0.042 [0.023, 0.064], SRMR = 0.017. These model fitting indexes showed this model fits well.

Table 1 presents the means and standard deviations of the demographic variables (gender, age) and research variables (perceived epidemic risk, security, perceived discrimination and depression symptoms). All the research variables demonstrated significant correlations. Specifically, perceived epidemic risk was negatively related to security (*r* = −0.25, *p* < 0.001) but positively related to perceived discrimination (*r* = 0.10, *p* < 0.001) and depression symptoms (*r* = 0.22, *p* < 0.001). Security was also negatively related to perceived discrimination (*r* = −0.23, *p* < 0.001) and depression symptoms (*r* = −0.54, *p* < 0.001). Additionally, perceived discrimination was positively related to depression symptoms (*r* = 0.13, *p* < 0.001).

### 3.2. Testing for the Moderated Mediation

Firstly, before adding other variables, we used the path analysis procedure in Mplus 8.0 to examine the predictive effect of perceived epidemic risk on depression symptoms. The results showed that perceived epidemic risk had a positive predictive effect on depression symptoms (*β* = 0.220, *SE* = 0.019, *p* < 0.001), verifying **Hypothesis 1**.

Secondly, security, perceived discrimination, and the product of perceived epidemic risk and perceived discrimination were added into the model (see Figure 2). The results showed that perceived epidemic risk could not significantly predict depression symptoms (*β* = 0.029, *SE* = 0.031, *p* > 0.05), perceived epidemic risk could significantly predict mediating variable security (*β* = −0.341, *SE* = 0.037, *p* < 0.001), and security also significantly predicted depression symptoms (*β* = −0.516, *SE* = 0.014, *p* < 0.001). The results from 1000 bootstrapping samples indicated that all of the simple path coefficients (path a, b) were statistically significant (*p* < 0.001, see Figure 2), with the bootstrapping 95% CI not including zero (see Table 2). Especially, the direct effect was not statistically significant (*p* > 0.05, see Figure 2). As a result, the model was completely mediated by perceived security. The total effect of perceived epidemic risk on depression symptoms was 0.205 (*p* < 0.001), and the proportion of the total indirect effect of depression symptoms on perceived epidemic risk estimated by perceived security was 85.85%. This verified **Hypothesis 2**.

Lastly, the results showed that the moderating effect of perceived discrimination on security (path c1) (*β* = −0.369, *SE* = 0.046, *p* < 0.001) and the interaction of perceived discrimination and perceived epidemic risk on security (path c3) (*β* = 0.219, *SE* = 0.059, *p* < 0.001) were statistically significant (see Figure 2, Table 2). This indicated that the perceived discrimination had a moderating effect between perceived epidemic risk and security. However, perceived discrimination did not moderate path b (path c2:*β* = −0.090, *SE* = 0.047, *p* > 0.05; path c4:*β* = 0.127, *SE* = 0.059, *p* < 0.05). This showed that perceived discrimination moderated perceived epidemic risk and depression symptoms by adjusting the impact of perceived epidemic risk on perceived security. This validated **Hypothesis 3**.

**Figure 3:**
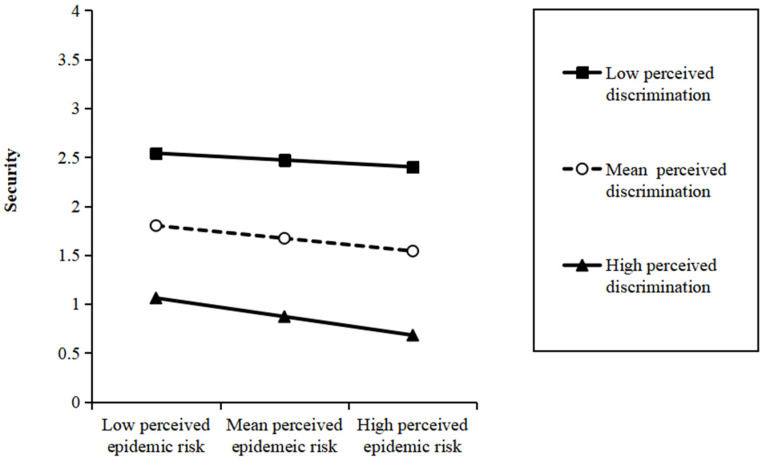
Interaction between perceived epidemic risk and perceived discrimination on security.

Next, we plotted the interaction pattern (see Figure 3). To be specific, the perceived epidemic risk was negatively associated with security when perceived discrimination was high. However, when perceived discrimination was low, the positive influence of perceived epidemic risk on security was weaker.

## 4. Discussion

COVID-19 has posed a threat to the life and health safety of people all over the world. Under the influence of such a sudden and strong health event, levels of individual mental health will be affected to varying degrees. The novel coronavirus pneumonia epidemic and depression symptoms are closely linked, according to research results from different countries. The COVID-19 pandemic has had a negative impact on mental health, such as increased anxiety and depression in the general population [65,66], healthcare workers [67], and teachers [68]. Furthermore, COVID-19 leads to more adverse psychological outcomes, such as anxiety and depression, in women than in men [68,69]. The purpose of this study was to clarify the mechanism of the relationship between perceived epidemic risk and depression symptoms by examining the mediating the effect of security and the moderating effect of perceived discrimination.

Consistent with previous studies, the current study results showed that perceived epidemic risk, security, and discrimination are associated with depression symptoms [14,37,47]. Firstly, this study found a significant correlation between individuals’ perception of epidemic risk and individual depression during the COVID-19 epidemic period, which supports **Hypothesis 1**. According to Lazarus’ transactional model of stress, when an individual thinks that their resources meet or exceed the needs of a situation, they will face a challenge [18]. Previous studies have found that risk information, as a negative stimulus, may cause threat, worry, and other negative emotions [70]. This shows that perceived risk is related to the individual’s emotional feelings. Some researchers have found that when individuals think that their risk of illness is low, they will show optimistic risk perception [71]. In other words, when individuals have a high level of risk perception, they will produce pessimistic and negative cognition. Extensive research has shown that most individuals experienced negative emotions such as depression during the epidemic period, such as in Pakistan 33–52% [12], Spain 12.7% [13], and the United States 64% [14]. The direct impact of perceived epidemic risk on depression symptoms indicates that individuals with high perceived epidemic risk may be more likely to feel negative emotions such as depression.

Secondly, the relationship between perceived epidemic risk and depression symptoms was found to be entirely mediated by perceived security, supporting **Hypothesis 2** of this study. Relevant studies show that when sense of security is high, the effect of perceived gender discrimination is mitigated [72]. Therefore, a higher perceived epidemic risk is associated with lower perceived security, and lower security leads to higher depression symptoms. The indirect impact of security shows that an individuals’ high level of risk perception can reduce their level of security in the environment; thus, in the context of an epidemic, an individual’s need for security increases. If the need for security is not met, an individual may experience more negative emotional reactions such as depression and anxiety. This high proportion of indirect effect indicates that security plays an important role in an individual’s depression symptoms.

Another finding of this study is that perceived discrimination has a moderating effect on the indirect impact path, supporting **Hypothesis 3**. At the same level of perceived epidemic risk, a high level of discrimination can increase the impact of epidemic risk on perceived sense of security, thus reducing an individual’s sense of security, resulting in the perception of greater levels of depression during the epidemic. This is consistent with previous studies [73]. Some researchers have found that workplace discrimination moderates the relationship between job satisfaction and job security. Attachment security is associated with low bias [74,75], indicating that bias and discrimination can follow the same pattern [76]. Discrimination perception, entailing an individual’s unfair treatment, has a significant predictive effect on neurosis [77], which is directly related to depression [78]. Lesbian, gay, transgender, queer, intersex, and other sexual and gender minorities are at higher risk of adverse mental health outcomes, such as suicidal ideation and depression [17,79], probably due to stigma and discrimination [80]. One systematic study estimated that the lifetime incidence of depression in this population was 1.5 times that of the general population [81]. It can be assumed that the perception of discrimination will lead to a lack of perceived security, which will prevent individuals from thinking positively. When an individual stops thinking positively, the continuous negative emotions resulting from discrimination surface, and other negative coping styles may further aggravate the individual’s anxiety and depression. Therefore, in the context of the COVID-19 epidemic, low discrimination can play a protective role for individuals. We can reduce this discrimination against individuals in order to alleviate the high level of perceived epidemic risk, promote individuals’ perceived security, and reduce their level of depression.

In the context of the COVID-19 epidemic, perceived epidemic risk and security directly affect the severity of depression. The government plays an important role in the intervention of people’s depression caused by COVID-19. However, if the government implements improper measures, people may perceive the epidemic risk as high and security as reduced, which may lead to or increase levels of depression. For example, the number of COVID-19 infections is not reported, and information about COVID-19 is not made public. If local governments do not intervene in COVID-19 discrimination, patients may experience high levels of perceived discrimination or stigma, leading to depression. Furthermore, depression was higher when the Government Response Stringency Index score was higher [82]. It is important for governments to take measures to protect people’s mental health in both areas. For example, first of all, the government can strengthen the publicity of epidemic prevention, encourage people to wear masks when traveling, popularize the knowledge of epidemic prevention, and reduce people’s panic from a scientific level. Secondly, the government can enable people to grasp the latest trends of the epidemic by disclosing the mortality and morbidity of the epidemic in the region. Finally, the government can explain how external stimuli stimulate depression in the epidemic situation, and establish a theoretical model. As pointed out by Wilson and Cleary (1995), identifying causal pathways that link different types of outcomes helps to optimize the design of interventions [83]. Only from understanding the potential correlation mechanisms between the influencing factors of depression can we formulate reasonable and effective measures to reduce depression, relieve psychological pressure, and provide the possibility for the development of mental health. Therefore, various national mental health institutions should take active and effective measures to improve people’s sense of security and reduce discrimination so as to protect people’s mental health fundamentally.

## 5. Strength and Limitations

Our research has several strengths. Firstly, according to this study, measures can be taken to reduce individual depression in terms of individual security and perceived discrimination. COVID-19 is harmful to people’s physical and mental health. From the perspective of individual psychology, this study is of great significance for preventing individual depression due to identifying the need to intervene in the perception of epidemic risk, sense of security, and perceived discrimination. It shows that, in the context of the COVID-19 epidemic, the impact of perceived epidemic risk on individuals’ depression is significant, and this impact can be completely explained by the effect of perceived security. In the above process, a high level of perceived discrimination can significantly increase the influence of individuals’ perceived epidemic risk on their perceived security. Our results reveal the potential mechanism between these psychological variables and depression. Additionally, this study has suggested some practical implications to aid mental health in the COVID-19 epidemic, such as reducing perceived discrimination.

Despite the above advantages, our research has the following limitations. Firstly, although the sample size of this study was ideal, the small number of male participants and the unbalanced gender proportion may limit the universality of our results. However, in the studies of other Chinese scholars, the ratio of women to men is also about 2 [3,11,15]. In this study, the results of the independent sample t-test showed that there was no significant gender difference in security (*p* = 0.056) and depression symptoms (*p* = 0.079). For the perceived epidemic risk, there was a significant gender difference (*p* = 0.000), and boys (*M* = 1.76) had significantly higher levels than girls (*M* = 1.67). There was also a significant gender difference in perceived discrimination (*p* = 0.000), and boys (*M* = 1.78) had significantly higher levels than girls (*M* = 1.59). The above results may be caused by the difference in the proportion of men and women involved in this study. Secondly, the nature and influencing factors of risk perception and depression are complex and not fully researched in the current study. Perceived discrimination can alleviate the negative impact of perceived epidemic risk on perceived security. However, whether it can regulate the effect of security on depression symptoms is still inconclusive. In the current study, we have omitted some variables, and thus future research should focus on exploring the relationship between perceived discrimination and other influencing variables. Finally, a cross-sectional study design was adopted in this study, which limits the interpretation of our results. Future research should be carried out with a longitudinal study controlling other variables that may affect the results to verify the current conclusions.

## 6. Conclusions

These findings shed light on the influence of perceived risk of the epidemic on depression symptoms in the context of the epidemic situation. We found that the level of epidemic risk perceived by the public changes their own sense of security, thereby increasing their own level of depression symptoms. Moreover, perceived discrimination moderates the mediating role of sense of security, which may help to develop targeted interventions. Based on the research results of other regions, it can be said that, in order to improve people’s mental health level during the epidemic, we can reduce people’s depression by improving people’s psychological perceived sense of security, which has important practical reference value for mental health protection in different countries and regions. In addition, for future research, we can further balance the composition of the sex ratio of the research subjects and adopt longitudinal investigation and research to improve our results.

## Figures and Tables

**Table 1:** Means, standard deviations, and intercorrelations of variables (*N* = 3443).

Variable	M	SD	1	2	3	4	5	6
(1) Gender	1.64	0.48	---					
(2) Age	26.25	9.30	−0.16 ***	---				
(3) Perceived epidemic risk	1.71	0.67	−0.06 ***	0.03	---			
(4) Security	3.63	0.93	0.03	0.03	−0.25 ***	---		
(5) Perceived discrimination	1.66	0.97	−0.09 ***	0.10 ***	0.10 ***	−0.23 ***	---	
(6) Depression symptoms	1.44	0.65	0.03	−0.04 **	0.22 ***	−0.54 ***	0.13 ***	---

Note. Gender was dummy coded as 0 = female and 1 = male. *p* > 0.05, ** *p* < 0.01, *** *p* < 0.001.

**Table 2:** Bootstrap mediation and mediated moderation effect (*N* = 3443).

	Point Estimate	Product of Confidence		BOOTSTRAP 1000 TIMES 95% CI	
		S.E.	Est/S.E.	Lower	Upper
Direct effect					
perceived epidemic risk → depression symptoms	0.029	0.031	0.960	−0.032	0.091
Indirect effect					
perceived epidemic risk → security → depression symptoms	0.176 ***	0.020	8.945	0.138	0.213
Total	0.205 ***	0.037	5.573	0.126	0.274
moderation effect					
perceived discrimination	−0.369 ***	0.046	−7.998	−0.461	−0.281
perceived epidemic risk X perceived discrimination	0.219 **	0.059	3.729	0.101	0.335

Note. *p* > 0.05, ** *p* < 0.01, *** *p* < 0.001.

## Data Availability

The datasets generated during and/or analyzed during the current study are available from the corresponding author on reasonable request.
